# Supplementary material for: DPP-4 inhibitors may improve the mortality of coronavirus disease 2019: A meta-analysis
Source: PLoS One. 2021 May 20;16(5):e0251916. doi: 10.1371/journal.pone.0251916 (PMC8136680; doi:10.1371/journal.pone.0251916)
Supplement: S1 File — (DOCX) [file pone.0251916.s002.docx]

| **Database** | **Search strategy** |
| --- | --- |
| PubMed | ((dipeptidyl peptidase-4 inhibitors) OR (Dpp4) OR (DPP-4) OR (saxagliptin) OR (alogliptin) OR (sitagliptin) OR (linagliptin) OR (vildagliptin)) AND ((COVID-19) OR (SARS Cov-2) OR (coronavirus) OR (2019 novel coronavirus)) |
| Embase | (‘dipeptidyl peptidase-4 inhibitors’ OR ‘Dpp4’ OR ‘DPP-4’ OR ‘saxagliptin’ OR ‘alogliptin’ OR ‘sitagliptin’ OR ‘linagliptin’ OR ‘vildagliptin’) AND (‘COVID-19’ OR ‘SARS Cov-2’ OR ‘coronavirus’ OR ‘2019 novel coronavirus’) |
| Web of science | ((dipeptidyl peptidase-4 inhibitors) OR (Dpp4) OR (DPP-4) OR (saxagliptin) OR (alogliptin) OR (sitagliptin) OR (linagliptin) OR (vildagliptin)) AND ((COVID-19) OR (SARS Cov-2) OR (coronavirus) OR (2019 novel coronavirus)) |
| Cochrane Library | (dipeptidyl peptidase-4 inhibitors or Dpp4 or DPP-4 or saxagliptin or alogliptin or sitagliptin or linagliptin or vildagliptin) AND (COVID-19 or SARS Cov-2 or coronavirus or 2019 novel coronavirus) |

# Full electronic search

Full electronic search performed in multiple international databases.

- PubMed
- Embase
- Web of Science
- Cochrane Library
