# Supplementary material for: DPP-4 inhibitors may improve the mortality of coronavirus disease 2019: A meta-analysis
Source: PLoS One. 2021 May 20;16(5):e0251916. doi: 10.1371/journal.pone.0251916 (PMC8136680; doi:10.1371/journal.pone.0251916)
Supplement: Attachment — Submitted filename: Response letter.docx [file pone.0251916.s003.docx]

Dear Reviewers,

We were pleased that the reviewers agreed that “the findings are of great interest”. Thank you for the constructive comments and suggestions; we found them helpful for improving our manuscript. We have modified the manuscript according to these suggestions. Our responses to the comments on the manuscript are provided below.

**Reviewer #1: DPP-4 Ace receptor are part of the receptor for viral entry. Studies from various European contries especially study from French Cohort has shown that the effect ACE inhibitor is neither harmful nor beneficial. Do they think same type of response from DPP-4 Inhibitor?**

Thank you for the constructive comments. We presume that you would like us to include the results of the study by the Carious team [1]. The authors mentioned in their article that a significant difference in the effect of ACE inhibitors on COVID-19 was not observed (OR=1.43 95% CI, 0.99-2.08), and the effects of DPP-4 inhibitors were not significantly different, but they tended to exert a protective effect (OR=0.85 95% CI, 0.55-1.32). Interestingly, when we combined their results with the results of the other included studies [2-4], we ultimately observed a significantly different protective effect (Figure 2). **Page-3 paragraph 2= DPP-4 are** **neutral agents but not hypoglycemic agents. Sentence need to be modified.**

Thank you for the constructive suggestion. We have modified this sentence to “DPP-4 inhibitors (DPP-4is), one of the new oral therapies for diabetes characterized by neutral weight and few adverse effects, is now used to improve insulin secretion as a treatment for T2DM” in the manuscript (lines 8 to 10 on page 3). **Page-4, para 2.1= We started article started from...…..= sentence needs to be modified.**

Thank you for the kind reminder. We have modified this sentence in the revised manuscript (line 12 to 13 on page 4). **Page-6, search results.. what was the probable cause of duplication?**

Thank you for the useful suggestion. When we searched different databases with the same keywords, the same documents or results are often retrieved. Here, we used Endnote to find duplicate results and avoid wasting effort in screening documents. **Page-9, paragraph 2 line3= DPP-4 should be replaced from DPP-4 inhibitor**

Thank you for the constructive suggestion. We have replaced DPP-4 with DPP-4 inhibitor in our revised manuscript (line 19 on page 8). **Page-9 Para3= Is this phenomena agent specific or non-specific?
Outcome of results should be mentioned properly not in a sketchy manner.**

We thank the reviewer for this constructive suggestion. DPP-4 degrades GLP-1, and GLP-1 exerts an anti-inflammatory effect, but researchers have not determined whether DPP-4is also inhibit the degradation of GLP-1 in patients with COVID-19. The anti-inflammatory effect is only our conjecture at present, and further proof is needed. We have added this explanation to the manuscript (lines 14-17 on page 9). **The stratification of Covid-19 positive patients should have been done by using various tools like modified APACHE-2 and SOFA,****MIMS etc.**

Thank you for the kind reminder. Unfortunately, since the available literature does not provide sufficient grouping information, we are unable to conduct this experiment based on this constructive suggestion.  **In Discussion- Inconclusive. Number of subjects very less. It should have been compared with SGLT-2 inhibitors with or without ACE inhibitors.**

Thank you for this constructive suggestion. We have added the control groups of our included researches in the Table 1. Unfortunately, due to the incomplete raw data of the included articles, it is unclear whether SGLT-2 inhibitors or ACE inhibitors is involved.

**Reviewer #2: The authors have conducted a meta-analysis of the existing studies on the effect of DPP4i use on COVID-19 mortality in T2DM patients. The findings are of great interest. However, the rationale of the study (SARS-CoV and SARS-CoV2 do not use DPP4 as the receptor, see Cell Study Markus Hoffmann, Hannah Kleine-Weber, Simon Schroeder, Nadine Krüger, Tanja Herrler, Sandra Erichsen, Tobias S. Schiergens, Georg Herrler, Nai-Huei Wu, Andreas Nitsche, Marcel A. Müller, Christian Drosten, Stefan Pöhlmann, SARS-CoV-2 Cell Entry Depends on ACE2 and TMPRSS2 and Is Blocked by a Clinically Proven Protease Inhibitor, Cell, Volume 181, Issue 2, 2020, Pages 271-280.e8,ISSN 0092-8674,**
[**https://doi.org/10.1016/j.cell.2020.02.052.,**](https://doi.org/10.1016/j.cell.2020.02.052.,) **Jean K Millet, Javier A Jaimes, Gary R Whittaker, Molecular diversity of coronavirus host cell entry receptors, FEMS Microbiology Reviews, 2020;, fuaa057,** [**https://doi.org/10.1093/femsre/fuaa057**](https://doi.org/10.1093/femsre/fuaa057)**),**
We sincerely appreciate this constructive suggestion. Taking into account the latest developments on the role of DPP-4is in the entry of COVID-19 into cells, as you suggested, we have corrected and updated our manuscript (lines 2-4 on page 2 and lines 5-12 on page 3). **the presentation of the data (writing of the results section) and therefore the discussion is not adequate**
Thank you for this constructive suggestion. We have modified the descriptions of the presentation of the data in the discussion section (line 5 on page 8). **The grammar and syntax are not up to Standard for smooth reading experience. In addition, there are spelling mistakes**

Thank you for this valuable advice; we have modified the whole manuscript carefully. Moreover, we have submitted our manuscript to American Journal Experts for language editing, and the entire manuscript has been revised.

**References**

1.	Cariou B, Hadjadj S, Wargny M, Pichelin M, Al-Salameh A, Allix I, et al. Phenotypic characteristics and prognosis of inpatients with COVID-19 and diabetes: the CORONADO study. Diabetologia 2020;63(8):1500-1515.doi:10.1007/s00125-020-05180-x

2.	Fadini GP, Morieri ML, Longato E, Bonora BM, Pinelli S, Selmin E, et al. Exposure to dipeptidyl-peptidase-4 inhibitors and COVID-19 among people with type 2 diabetes: A case-control study. Diabetes, obesity & metabolism 2020;22(10):1946-1950.doi:10.1111/dom.14097

3.	Solerte SB, D'Addio F, Trevisan R, Lovati E, Rossi A, Pastore I, et al. Sitagliptin Treatment at the Time of Hospitalization Was Associated With Reduced Mortality in Patients With Type 2 Diabetes and COVID-19: A Multicenter, Case-Control, Retrospective, Observational Study. Diabetes care 2020;43(12):2999-3006.doi:10.2337/dc20-1521

4.	Strollo R, Maddaloni E, Dauriz M, Pedone C, Buzzetti R, Pozzilli P. Use of DPP4 inhibitors in Italy does not correlate with diabetes prevalence among COVID-19 deaths. Diabetes research and clinical practice 2021;171:108444.doi:10.1016/j.diabres.2020.108444
