# Supplementary material for: DPP-4 inhibitors may improve the mortality of coronavirus disease 2019: A meta-analysis
Source: PLoS One. 2021 May 20;16(5):e0251916. doi: 10.1371/journal.pone.0251916 (PMC8136680; doi:10.1371/journal.pone.0251916)
Supplement: Attachment — Submitted filename: PLOS ONE REVIEW.docx [file pone.0251916.s004.docx]

No Changes required. Manuscript accepted after revision
