# Supplementary material for: DPP-4 inhibitors may improve the mortality of coronavirus disease 2019: A meta-analysis
Source: PLoS One. 2021 May 20;16(5):e0251916. doi: 10.1371/journal.pone.0251916 (PMC8136680; doi:10.1371/journal.pone.0251916)
Supplement: Attachment — Submitted filename: Response letter.docx [file pone.0251916.s005.docx]

Dear Reviewers,

We were pleased that the reviewers agreed that “the findings are of great interest”. Thank you for the constructive comments and suggestions; we found them helpful for improving our manuscript. We have modified the manuscript according to these suggestions. Our responses to the comments on the manuscript are provided below.

**Reviewer #2: The authors have addressed some of my comments.
However, they need to address additional concerns that have arisen due to oversight and/or not adequately addressing them on the revision.

1. Last para on Pg8 of the manuscript, did the authors mean DPP4 or** **DPP4 inhibitors**

Thank you for the constructive comments. We have replaced the “DPP4 inhibitors” with “DPP4” in the revised manuscript (lines 19 on page 8). **2. Reference 27 is not directly related to ARDS although it could be extrapolated from an LPS-induced mouse model. The way it is written, it seems that Sitagliptin has proven efficacy against ARDS**

Thank you for the useful suggestion. We have redescribed this sentence as you suggested in our revised manuscript (lines 2 to 3 on page 9).
